# A Neurocomputational Account of How Inflammation Enhances Sensitivity to Punishments Versus Rewards

**DOI:** 10.1016/j.biopsych.2015.07.018

**Published:** 2016-07-01

**Authors:** Neil A. Harrison, Valerie Voon, Mara Cercignani, Ella A. Cooper, Mathias Pessiglione, Hugo D. Critchley

**Affiliations:** a Department of Psychiatry, Brighton and Sussex Medical School, Brighton; b Sackler Centre for Consciousness Science, University of Sussex Brighton; c Sussex Partnership National Health Service Foundation Trust, Brighton; d Department of Psychiatry, University of Cambridge, Cambridge; e Cambridgeshire and Peterborough National Health Service Foundation Trust, Cambridge; f Clinical Imaging Sciences Centre, Brighton and Sussex Medical School, University of Sussex, Brighton, United Kingdom; g Motivation, Brain & Behavior Lab, Brain & Spine Institute, Pitié-Salpêtrière Hospital, Paris, France

**Keywords:** Depression, Imaging, Inflammation, Insula, Reward, Striatum

## Abstract

**Background:**

Inflammation rapidly impairs mood and cognition and, when severe, can appear indistinguishable from major depression. These sickness responses are characterized by an acute reorientation of motivational state; pleasurable activities are avoided, and sensitivity to negative stimuli is enhanced. However, it remains unclear how these rapid shifts in behavior are mediated within the brain.

**Methods:**

Here, we combined computational modeling of choice behavior, experimentally induced inflammation, and functional brain imaging (functional magnetic resonance imaging) to describe these mechanisms. Using a double-blind, randomized crossover study design, 24 healthy volunteers completed a probabilistic instrumental learning task on two separate occasions, one 3 hours after typhoid vaccination and one 3 hours after saline (placebo) injection. Participants learned to select high probability reward (win £1) and avoid high probability punishment (lose £1) stimuli. An action-value learning algorithm was fit to the observed behavior, then used within functional magnetic resonance imaging analyses to identify neural coding of prediction error signals driving motivational learning.

**Results:**

Inflammation acutely biased behavior, enhancing punishment compared with reward sensitivity, through distinct actions on neural representations of reward and punishment prediction errors within the ventral striatum and anterior insula. Consequently, choice options leading to potential rewards were less behaviorally attractive, and those leading to punishments were more aversive.

**Conclusions:**

Our findings demonstrate the neural mediation of a rapid, state-dependent reorientation of reward versus punishment sensitivity during inflammation. This mechanism may aid the adaptive reallocation of metabolic resources during acute sickness but might also account for maladaptive, motivational changes that underpin the association between chronic inflammation and depression.

Inflammation rapidly reorients motivational state; pleasurable activities are avoided, sensitivity to negative stimuli is enhanced, and feelings of depression, fatigue, and irritability are common (1, 2). Mediated by the host immune response, this motivational shift efficiently prioritizes whole organism responses to the infecting agent (1, 2). However, how inflammation mediates these rapid shifts in behavior currently remains unclear.

To address this, we used computational modeling of a reinforcement-learning task to dissect effects of inflammation on reward- and punishment-related decision-making processes. Importantly, this approach allows computation of hidden prediction error signals (δ), the teaching signal embodied in contemporary computational reinforcement learning theory, critical to updating estimates of the value of available options and consequent biasing of behavioral choice (3). Data from rodents and primates suggest that midbrain dopaminergic cells may provide this teaching signal at least in the context of reward learning (4, 5, 6, 7), with actions on corticostriatal synaptic efficacy providing a mechanism for flexible reward learning and behavioral optimization. Dopamine-dependent modulation of striatal reward prediction error has also been linked to human reinforcement learning to reward (8). It is therefore noteworthy that inflammation has been observed to modulate striatal dopamine uptake (9) and efflux (10), as well as ventral striatal responses to both reward outcome (9) and cues predicting reward (11), suggesting that the rapid changes in reward-related behavior induced by inflammation may be mediated via an action on striatal reward prediction error encoding.

However, behavioral effects of inflammation are not limited to changes in reward-related behavior. In both rodents and humans, experimentally induced inflammation has also been shown to enhance sensitivity to punishment, at least when experienced as musculoskeletal pain (12, 13). Though proinflammatory mediators can sensitize peripheral nociceptors (14), lipopolysaccharide-evoked hyperalgesia does not typically develop until 3 hours (13, 14, 15), suggesting a likely role for central sensitization processes, an interpretation supported by the characteristic pattern of mechanical but not thermal hyperalgesia (16). Interestingly, studies investigating reinforcement learning to punishment have identified a punishment specific prediction error signal within insula cortex (8, 17), a region implicated in signaling a range of aversive events (18, 19, 20, 21) including pain (22) and peripherally induced inflammation (23, 24, 25). Correspondingly, patients with insula lesions show impairment in punishment but not reward-based learning (26). Whether previously observed actions of inflammation on insula reactivity additionally modulate punishment prediction error signals, proving a mechanism for enhancing sensitivity to punishment, was a second focus of the current study.

To investigate the behavioral and brain mechanisms mediating this inflammation-induced motivational reorientation (expressed as enhanced punishment sensitivity and simultaneously impaired reward sensitivity), we studied 24 healthy individuals (18 during functional magnetic resonance imaging [fMRI]) on two separate occasions, one 2.5 to 3.5 hours after a standard inflammatory challenge (typhoid vaccination) and one 2.5 to 3.5 hours after control (saline injection). We applied a reinforcement-learning model to a probabilistic learning task and restricted our primary hypotheses to ventral striatum and insula regions previously shown to encode reward and punishment prediction error, respectively (8). We hypothesized that inflammation would impair sensitivity to gains (win £1) (manifest as an acute reduction in ventral striatal positive δ and a consequent reduction in the propensity to choose the most rewarding action on a reinforcement-learning task) and simultaneously enhance sensitivity to punishment (observed as an enhancement in insula negative δ on loss trials and a consequent increase in the propensity to avoid the punishing [lose £1] choice).

## Methods and Materials

### Inclusion and Exclusion Criteria

Twenty-four healthy nonsmokers (9 male subjects, mean 27.6 ± 7.0 years) were recruited and screened for relevant physical or psychiatric illness. One was later excluded after failure to complete the second scanning session. Volunteers who had received typhoid vaccine within 3 years or other vaccine within 6 months were excluded. All were medication free and rated their general health as good, very good, or excellent. Participants were advised to not consume alcohol, avoid high-fat meals, and refrain from excessive exercise for 24 hours before testing and avoid nonsteroidal anti-inflammatory drug medications, steroids, and antibiotics for 7 days before testing. Written informed consent was obtained after complete description of the study and study procedures were approved by the Brighton-East National Research Ethics Committee.

### Study Design

We adopted a randomized, repeated-measures, cross-over design with both participant and researcher blind to intervention. Participants underwent two separate testing sessions 7 days apart. In the first session, participants were randomly assigned to one of two experimental conditions (typhoid vaccine or saline injection) with 12 participants receiving typhoid vaccination in the first session. Baseline blood samples were taken and then injections of .025 mg Salmonella typhi capsular polysaccharide vaccine or .5 mL normal saline placebo were administered intramuscularly into the deltoid muscle. Behavioral testing was performed 2.5 to 3.5 hours after injection in a 60-minute session (23); 18 participants completed testing during fMRI and 6 completed testing in a behavioral testing suite. Immediately after testing, a second blood sample was taken for repeat cytokine measurement. Body temperature and Profile of Mood States (POMS) questionnaire with four extra items (fever, aching joints, nausea, and headache) added to assess somatic symptoms associated with mild infection (27) were completed at baseline and after 3.5 hours. The second testing session was identical except that participants received the alternate injection (i.e., typhoid vaccination if they previously received saline and vice versa).

### Reinforcement Learning Task

**Figure 1:**
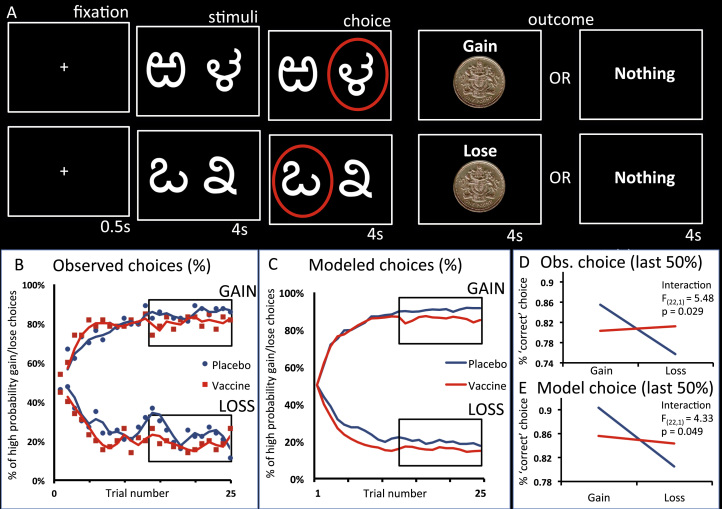
Experimental task and behavioral responses. **(A)** Participants chose between stimulus pairs from three conditions: gain (upper images), loss (lower images), and neutral (not shown) associated with the corresponding pairs of outcomes: gain £1/nothing, lose £1/nothing, and look £1/nothing. The two stimuli forming each stimulus pair had reciprocal probabilities (.8/.2 and .2/.8) of receiving the corresponding outcome. For example, in the gain condition, one of the stimulus pairs had an 80% chance of winning £1 and a 20% chance of winning nothing; the other option had a 20% chance of winning £1 and an 80% change of winning nothing. Stimulus pairs were presented randomly, with the high probability win/loss/look stimulus presented on the right on 50% of trials and on the left on 50% of trials. **(B)** Observed behavioral choices for gain and loss conditions following placebo (blue) or typhoid vaccine induced inflammation (red). The learning curves (moving average) depict trial by trial the percentage of times participants chose the correct stimulus (probability = .8 of winning £1) upper graph and the incorrect stimulus (probability = .8 of losing £1). **(C)** Modeled behavioral choices for placebo (blue) and inflammation (red). The learning curves represent the probabilities predicted by the computational model. **(D)** Proportion of the last 50% of trials in which participants chose the correct stimulus for both gain (left) and loss (right) conditions. **(E)** Modeled behavioral choices for the proportion of the last 50% of trials in which participants chose the correct stimulus. Obs., observed.

Participants completed three runs of the same instrumental learning task, each using three new pairs of abstract stimuli on each testing session (Figure 1A). Each pair of stimuli (gain, loss, neutral) was associated with a pair of outcomes (gain £1/nil, lose £1/nil, look £1/nil), and the two stimuli corresponded to reciprocal probabilities (.8/.2 and .2/.8). On each trial, one pair was randomly presented with the two stimuli presented left and right of a central fixation cross; relative positions were counterbalanced across trials. The participant chose the right-sided stimulus with a button press (go response) and the left-sided stimulus with an absence of a response (no-go response). The choice was then circled in red and the outcome displayed on the screen after a 4-second delay. To maximize winnings and minimize losses, participants had to use trial and error to learn stimulus-outcome associations. They were told that they would be remunerated their winnings, though all left with the same fixed amount. Effects of inflammation on behavioral performance were assessed using repeated-measures analysis of variance (ANOVA).

### Computational Model

A standard algorithm of action-value learning that combines the Rescorla-Wagner learning rule (which updates chosen option values in proportion to reward prediction errors) and a softmax decision rule (which estimates choice probability as a sigmoid function of the difference between the two option values Q_a_ and Q_b_) (8, 28) was fitted to the observed behavior. For each pair of stimuli (A and B), the model used each individual’s sequences of choices and outcomes to estimate the expected values of choosing A (Q_A_) and B (Q_B_). Expected values (Q_A_ and Q_B_) were initialized at zero and the value of stimulus chosen at each trial (e.g., A) was updated according to the rule Q_A_(t + 1) = Q_A_(t) + α × δ(t), with outcome prediction error δ(t) defined as the difference between the actual and expected outcome, δ(t) = R(t) – Q_A_(t). Given the expected values, the probability of the observed choice was estimated using the softmax rule P_A_(t) = exp(Q_A_(t) / β) / {exp[Q_A_(t) / β] + exp[Q_B_(t) / β]}. The free parameters alpha (learning rate), beta (temperature), and R (subjective value) were adjusted to maximize the likelihood of each participant’s observed choices under the model.

### Cytokine Analysis

Blood (10 mL) was collected in ethylenediaminetetraacetic acid vacutainer tubes (Becton Dickinson and Company, Franklin Lakes, New Jersey) and centrifuged at 1250*g* for 10 minutes; then plasma was removed, aliquoted, and frozen at −80°C. Plasma interleukin-6 (IL-6) was assessed using high-sensitivity enzyme-linked immunosorbent assays (R&D Systems, Abingdon, United Kingdom). The limit of detection of the IL-6 assay was .039 pg/mL, with intra-assay and interassay coefficients of variation of 7.4% and 7.8%. Cytokine analysis was performed using repeated-measures ANOVA in SPSS 22 (IBM Corp., Armonk, New York).

### Image Acquisition and Analysis

T2*-weighted echo planar images (EPIs) were acquired on a 1.5T Siemens Avanto magnetic resonance scanner equipped with a 12-channel head coil (Siemens Healthcare, Erlangen, Germany) using a −30° tilted acquisition to reduce orbitofrontal dropout (29). Each volume provided whole-brain coverage (40 interleaved ascending 2 mm slices with 1 mm interslice gap, echo time 40 ms: repetition time 3.3 s, spatial resolution 3 mm^3^). High-resolution inversion-recovery echo planar images were additionally acquired, segmented, and then normalized in SPM8 (Wellcome Trust Centre for Neuroimaging, Institute of Neurology, United College London, United Kingdom; http://www.fil.ion.ucl.ac.uk/spm) to aid group-level anatomical localization. EPIs were analyzed in an event-related manner using SPM8. Preprocessing consisted of spatial realignment, segmentation, and normalization of the mean EPI image to a standard EPI template and then spatial smoothing with an 8 mm full-width at half maximum Gaussian kernel. Subject-specific realignment parameters were modeled as covariates of no interest to correct for motion artifacts. Stimulus and outcome onsets were modeled as separate delta functions and convolved with a canonical hemodynamic response function. Prediction errors and Q-values calculated by the computational model were used as additional regressors that parametrically modulated outcome and cue onsets, respectively. Linear contrasts of regression coefficients were computed at the individual subject level and then taken to group level repeated-measures ANOVA (factors: inflammation [vaccine, placebo], condition [gain, loss]). Activation maps for reward prediction error (rPE) and punishment prediction error (pPE) in ventral striatum and anterior insula reported in the original article (8) using this task were obtained and used as region of interest masks. All group-level statistical parametric maps are reported with a whole-brain or region of interest familywise error correction threshold of *p* < .05.

## Results

### Participant Characteristics

Behavioral outcomes were derived from 24 healthy nonsmokers (9 male subjects, mean 27.6 ± 7.0 years) screened for a history of relevant physical or psychiatric illness. Of these, 18 were scanned (one did not complete the second scanning session due to technical difficulties). All were medication free and rated their general health as good, very good, or excellent.

### Response to Typhoid Vaccination

**Figure 2:**
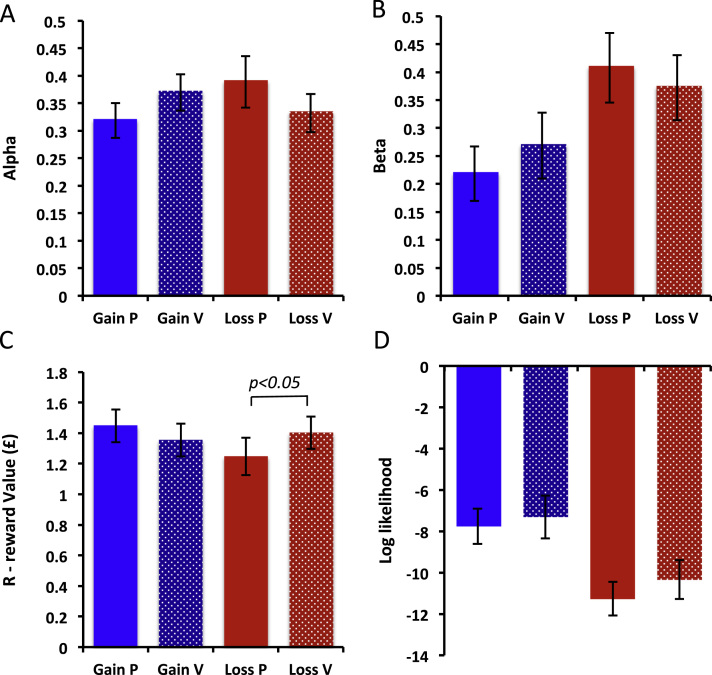
Effects of vaccination on model parameters. Behavioral outcomes for model parameters: **(A)** alpha (learning rate), **(B)** beta (choice randomness or temperature), **(C)** R (subjective value), and **(D)** log likelihood of model fit. Data are represented as solid bars for gain and shaded bars for loss conditions. Higher values represent faster learning, greater choice randomness, reward (and punishment) subjective value, and model fit, respectively. Error bars represent standard error of the mean.

Typhoid vaccination evoked a robust inflammatory response with an approximately 250% increase in plasma IL-6 from mean (± SE) .89 ± .23 pmol/L at baseline to 2.17 ± .26 pmol/L at 3½ hours (*t*_22_ = 5.21, *p* < .001) (Figure 2A). The placebo condition evoked a smaller nonsignificant rise in IL-6 from .73 ± .15 pmol/L at baseline to .94 ± .16 pmol/L at 3.5 hours (*t*_15_ = 1.60, *p* = .13). This was confirmed by a significant treatment (inflammation, placebo) by sample (baseline, 3.5 hours) interaction for IL-6 (*F*_1,22_ = 17.13, *p* < .001).

There was no significant effect of vaccination on core body temperature (treatment × sample interaction: *F*_1,22_ = .81, *p* = .38) (Figure 2B) or somatic symptoms (interaction: *F*_1,22_ = .28, *p* = .60), confirming that effects were not driven by pain, temperature, or subjective discomfort. POMS measured fatigue significantly increased following inflammation (time × inflammation interaction: *F*_1,22_ = 5.02, *p* = .036). Though there was a larger drop in POMS total mood score in the inflammation compared with placebo condition (4.8 vs. 2.2 points), this was not statistically significant (*F*_1,22_ = 1.20, *p* = .28).

### Behavioral Outcomes

Inflammation was associated with a shift in reward versus punishment sensitivity, expressed as reduced selection of high probability reward, yet increased avoidance of high probability punishment stimuli (Figure 1B). This was supported by a significant inflammation (placebo, vaccine) by valence (reward, punishment) interaction (*F*_1,22_ = 5.48, *p* = .029) (Figure 1D). Of note, post hoc *t* tests for reward and punishment conditions were *p* = .195 and *p* = .071, respectively, indicating that inflammation induced a relative increase in sensitivity to punishment versus reward. Importantly, there was no significant main effect of inflammation or inflammation by valence interaction for go versus no-go responses, confirming equal task engagement across conditions (*p* > .10), and no significant main effect of session or session by condition (reward, punishment) interaction (*F*_1,22_ = .32, *p* = .58, and *F*_1,22_ = .63, *p* = .44, respectively). There was no significant main effect of time (session 1/session 2) or time by condition (gain/lose) interaction (*F*_1,22_ = .50, *p* = .49, and *F*_1,22_ = 1.26, *p* = .27, respectively).

To analyze this effect of inflammation on reward versus punishment sensitivity in more detail, we next fitted our reinforcement-learning model (3) to the observed choices. The three free model parameters, learning rate (α), choice randomness (β), and subjective value (R) were adjusted to optimally fit the model to the learning curves and maximize the likelihood of the observed choices. This was done separately for gain and loss conditions under both placebo and inflammation for each participant. The adjusted free parameters were then tested for condition effects (inflammation/placebo, reward/punishment) in repeated-measures ANOVAs.

Analysis of the model data confirmed the inflammation (placebo, vaccine) by valence (reward, punishment) interaction observed in the behavioral responses (*F*_1,22_ = 4.33, *p* = .049) (Figure 1C, E). Interestingly, inflammation was not associated with a change in either learning rate (α) or choice randomness (β) (*F*_1,22_ = 3.258, *p* = .082, and *F*_1,22_ = 1.781, *p* = .19, respectively) (Figure 2C, D). However, it was associated with a change in subjective value (R) (inflammation × valence interaction: *F*_1,22_ = 4.694, *p* = .041; Figure 2E), specifically an increase in the (negative) subjective value of the punished stimulus (paired *t*_22_ = −2.107, *p* = .047). There was no significant effect on the subjective value of the rewarded stimulus (paired *t*_22_ = .938, *p* = .359). Of note, participant behavior was equally well modeled across placebo and vaccine conditions (mean log likelihood = −9.17; interaction: *F*_1,22_ = .246, *p* = .624; Figure 2F).

### Imaging

**Figure 3:**
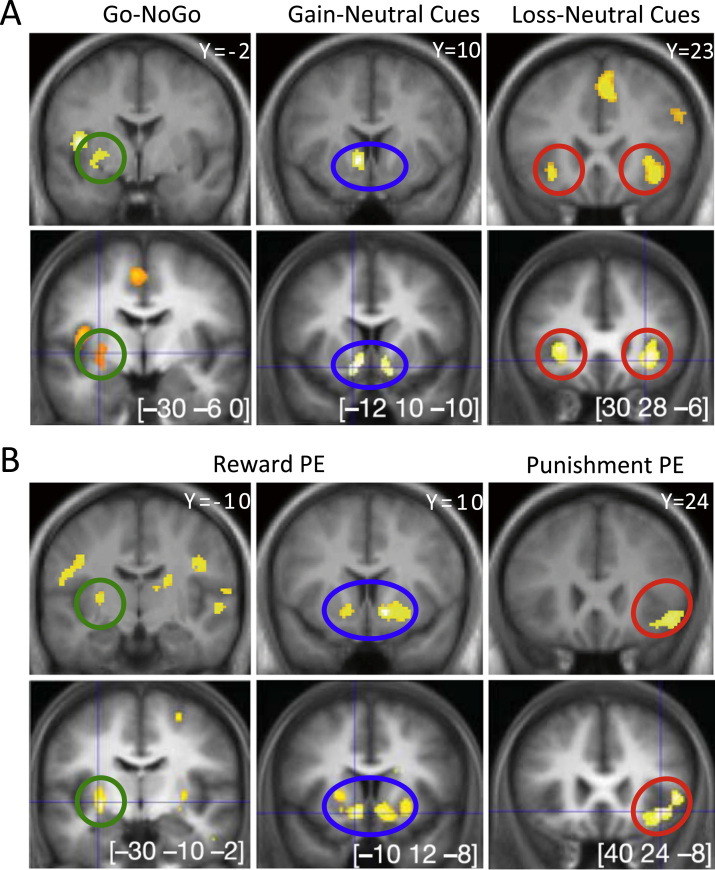
Brain regions correlating with reward and punishment cues and prediction error. Upper rows of both panels denote data from the current study at an uncorrected statistical threshold of *p* < .001. Lower rows show comparable contrasts from Pessiglione *et al*. (8). **(A)** Statistical parametric maps resulting from the main contrasts between stimuli conditions. Go and NoGo refer to stimuli requiring or not requiring a button press to get the optimal outcome. Gain, neutral, and loss correspond to the different pairs of stimuli. Activations are shown on slices comparable with Pessiglione *et al*. (8) located in the posterior putamen (green), left ventral striatum (blue), and bilateral insula (red). **(B)** Brain activity correlated with prediction errors (PE) derived from the computational model. Reward prediction errors (positive correlation) are shown across both gain and loss conditions (left and center panels); punishment prediction errors (negative correlation) are found in the loss condition alone (right panel). As above, activations are shown on slices comparable with Pessiglione *et al*. (8) located in the posterior putamen (green), left ventral striatum (blue), and bilateral insula (red). [Reproduced with permission from Pessiglione *et al*. (8).]

Modeling of gain versus neutral (look £1) cues was associated with significant activation within ventral striatum and left posterior putamen (Figure 3A) and loss versus neutral cues with bilateral ventral striatum and insula activation (Figure 3B; Supplemental Table S1), as described previously for this task in an independent population (8). We next used the reinforcement-learning model to extract trial by trial δ and predicted outcome, which were then used as parametric modulators of outcome and stimulus phases, respectively. Examination of the representation of outcome prediction error across both conditions (placebo, inflammation) demonstrated positive correlation with bilateral ventral striatum activity with an additional negative correlation with punishment prediction error in the left insula (Figure 3; Table 1), as previously reported with this task.

**Figure 4:**
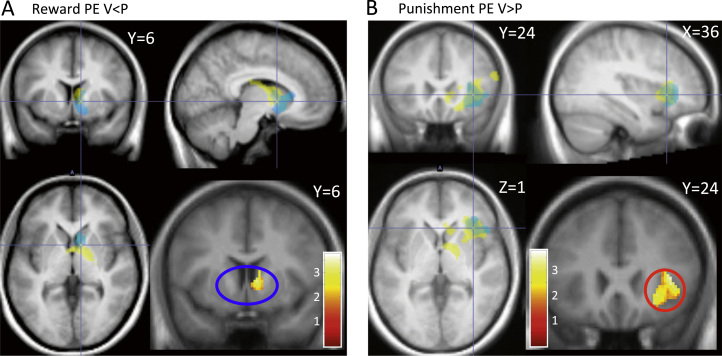
Effects of inflammation on ventral striatal and insula responses to reward and punishment prediction error (PE). **(A)** Bottom right panel: right ventral striatal region demonstrating significantly reduced correlation with reward prediction error following inflammation (compared with placebo). Remaining panels illustrate the same contrast (yellow) overlaid on the right ventral striatal region of interest mask (correlation with reward prediction error) from Pessiglione *et al*. (8) (cyan). **(B)** Bottom right panel: right insula region demonstrating significantly increased correlation with punishment prediction error following inflammation (compared with placebo). Remaining panels illustrate the same contrast (yellow) overlaid on the right insula region of interest mask (correlation with punishment prediction error) from Pessiglione *et al*. (8) (cyan). P, placebo; V, vaccine.

To further investigate the basis of the behavioral effects of inflammation, specifically increased sensitivity to punishment compared with reward, we next investigated effects of inflammation on ventral striatal and insula encoding of reward and punishment prediction error. Ventral striatum and insula regions of interest were first defined using clusters correlating with reward and punishment prediction error in an independent population (8). We then investigated effects of inflammation on reward and punishment prediction error within each region using the contrasts vaccine < placebo and placebo > vaccine, respectively. This demonstrated a significant reduction in ventral striatal encoding of reward prediction error following inflammation and conversely a significant increase in right insula encoding of punishment prediction error (Figure 4; Supplemental Table S2). Bayesian model selection (Supplement) supported mediation via actions on prediction error rather than outcome value (Supplemental Figure S1).

## Discussion

Theories of instrumental learning highlight a central role for prediction error signals in updating the values associated with available choices, aiding learning from success and failure and ultimately improving future decisions (30). Using a probabilistic instrumental learning task, we showed that experimentally induced inflammation significantly enhances sensitivity to punishments versus rewards. Modeling of individual choices using our reinforcement-learning model accurately reflected this pattern of behavioral effects and demonstrated a significant interaction between inflammation and model parameters for the subjective value of rewards versus punishments. Across conditions, we replicated and extended previous findings of correlations between ventral striatal and anterior insula activity and computationally determined reward (rPE) and punishment (pPE) prediction errors, respectively. However, we also showed that both were significantly modulated by inflammation, with inflammation prompting a significant reduction in the encoding of rPE within ventral striatum and a converse enhancement of insula encoding of pPE. Bayesian model selection (Supplement) further supported this mechanistic interpretation. These findings suggest that actions of inflammation on ventral striatal and insula regions encoding rPE and pPE together mediate the motivational reorientation characteristic of sickness behaviors (1, 2), differentially modulating how values associated with available choices are updated and ultimately enhancing sensitivity to punishments compared with rewards. They also provide further evidence for differential neural encoding of reward and punishment prediction error signals in humans.

Impairment in reward-related behavior is a core feature of the motivational reorientation characteristic of sickness behaviors and can be indexed in animals by reduced saccharin preference (31, 32, 33), anhedonia (2), and reduced rewarding electrical self-stimulation (10, 34). Previous human fMRI studies note inflammation-induced reductions in ventral striatal reactivity to both reward cues (11) and reward outcomes (9). Our fMRI data support and develop this literature by suggesting that impairments in reward-related behavior, which can be observed within hours of inflammatory challenge, may be mediated via specific actions on ventral striatal rPE encoding. Computational analyses captured this shift in plateau response across gain and loss conditions as a significant condition (gain, loss) by inflammation (vaccine, placebo) interaction for the subjective value of rewards compared with punishments. Nevertheless, it should be noted that, unlike the loss learning condition, this reduction in reward magnitude did not reach statistical significance for post hoc *t* test (*p* > .05). Further, though our behavioral data demonstrated a significant increase in relative sensitivity to punishments compared with rewards, there was no statistically significant reduction in reward (or punishment) sensitivity per se. Together, these data reveal that ventral striatal encoding of rPE, considered critical for reward learning, is sensitive to inflammatory state and affords one element of an efficient mechanism for the rapid reorientation of behavior in the face of an acute infection.

Though we did not measure dopamine activity directly, a similar reduction in striatal reward prediction error magnitude and propensity to choose the most rewarded action has previously been reported on this task after haloperidol (a dopamine receptor 2 antagonist) (8). This suggests that our observed changes in striatal prediction errors were likely mediated by actions of inflammation on dopamine release. It is therefore noteworthy that inflammation has also been linked to altered nucleus accumbens dopamine efflux in rodents (10) and reduced presynaptic dopamine synthesis or release in humans (9). Supporting this, monkeys showing behavioral impairment after inflammatory challenge with lipopolysaccharide exhibit significantly lower cerebrospinal fluid concentrations of the dopamine metabolite homovanillic acid (35). How inflammation modulates dopamine function is currently unclear. However, individual cytokines such as interferon-alpha have been shown to inhibit dopamine synthesis by reducing central nervous system tetrahydrobiopterin, an essential cofactor for tyrosine hydroxylase, the rate-limiting step in dopamine synthesis (36). Inflammation can also decrease synaptic dopamine by increased expression of the monoamine reuptake transporter (36, 37, 38, 39). Inflammation may further influence dopamine neurotransmission via activation of the tryptophan-degrading enzyme indoleamine 2,3-dioxygenase and resultant formation of neurotoxic kynurenine metabolites (2, 37).

Inflammation significantly enhanced sensitivity to punishments compared with rewards, suggesting a coordinated biasing of behavior toward avoidance of punishment yet decreasing sensitivity to reward. Computational analysis of the loss task revealed a distinct effect of inflammation, with greater avoidance of the punishing option (reflected as a lower plateau) specifically captured by a greater (negative) punishment subjective value. This was also reflected in the larger effect size of the right anterior insula correlation with negative pPE. Increasing pPE is one way to increase the subjective value of punishment, theoretically aiding discrimination of the two cues. This may serve as the computational mechanism by which the anterior insula drives the improvement in avoidance behavior. This is in line with theories proposing that brain areas involved with somatic affective representations (including inflammation) are causally responsible for making a choice (24, 40, 41, 42). This characteristic pattern of behavioral change is noteworthy as it complements an earlier study showing impaired sensitivity to punishment (with a higher plateau) in patients with selective insula lesions (26). Interestingly, it also suggests that relative sensitivity to reward versus punishment is a state rather than a trait-dependent attitude, flexibly enhancing loss minimization in the context of a threat to the organism (such as an infection) yet maximizing responses to gains when in good health.

Bayesian model selection suggested that pPE (as opposed to punishment outcomes) drove effects observed within the whole anterior insula region of interest, including the subregion showing sensitivity to inflammation. However, rPE only drove effects for the discrete ventral striatal subregion that showed sensitivity to inflammation, with effects within other regions of the ventral striatum being driven more by reward outcome. Interestingly, this region is consistent with effects of interferon on reward outcomes (9) but lies slightly more dorsal to a region previously shown to be sensitive to lipopolysaccharide-induced effects on reward cues (11).

Low-level systemic inflammation similar to that induced using the typhoid challenge model is increasingly implicated in the etiology of depression (2, 43), a condition itself characterized by impaired sensitivity to reward yet increased sensitivity to punishment (13, 44). Indeed, one in three patients given weekly injections of the pro-inflammatory cytokine interferon-alpha for hepatitis C develop major depression (45). Dysfunctional responses to negative feedback were among the earliest cognitive changes observed in depression, as predicted by models of learned helplessness (46). More recently, meta-analysis of computationally modeled reinforcement learning tasks has reported, similar to our own findings, a selective reduction in subjective reward value rather than reward learning rate in individuals with depression or a past history of depression (47). Relatively selective actions on reward/punishment magnitude, rather than learning rate or choice temperature, have also been reported following dopamine manipulation and insular damage (8, 26). Our findings of a rapid cognitive adaptation following inflammation heightening relative sensitivity to punishment versus reward raise the intriguing possibility that while this may be beneficial in the context of an infective challenge when metabolic resources are diverted to fighting the infecting organism, when chronic, they may predispose to developing the maladaptive changes in motivation observed in depression.

## Figures and Tables

**Table 1:** Significant Clusters Correlating With Reward and Punishment Prediction Error

Side	Region	Coordinates	*Z* Score	k	*p*	FWE (ROI)
Reward Prediction Error						
L	Ventral striatum	[−16 8 −8]	3.97	142	<.001	.063 (.006)
R	Ventral striatum	[12 8 −8]	5.01	232	<.001	.007 (.001)
L	Posterior putamen	[−26 −6 8]	4.12	118	<.001	.118 (.004)
R	Sensory-motor	[6 −32 68]	5.05	520	<.001	.001
R	STS	[56 −8 0]	4.76	292	<.001	.002
R	Inferior parietal	[52 −28 40]	4.58	254	<.001	.004
R	Cerebellum	[42 −66 −42]	4.41	321	<.001	.001
R	Occipital pole	[24 −100 12]	4.32	280	<.001	.003
Punishment Prediction Error						
R	Anterior insula	[42 −28 −10]	4.33	275	<.001	.002 (.023)
Mid	Striate cortex	[4 −78 −6]	6.85	1435	<.001	.001
L	Fusiform	[−24 −48 −14]	5.17	198	<.001	.013
R	Fusiform	[24 −64 −8]	5.01	456	<.001	.001
R	TPJ	[62 −40 28]	4.81	216	<.001	.009

Only clusters surviving whole brain or region of interest (reported in brackets) familywise error correction are reported. k denotes cluster extent; [x y z] are Montreal Neurological Institute coordinates.

FWE, familywise error; L, left; R, right; ROI, region of interest; STS, superior temporal sulcus; TPJ, temporoparietal junction.
