# Supplementary material for: A Neurocomputational Account of How Inflammation Enhances Sensitivity to Punishments Versus Rewards
Source: Biol Psychiatry. 2016 Jul 1;80(1):73–81. doi: 10.1016/j.biopsych.2015.07.018 (PMC4918729; doi:10.1016/j.biopsych.2015.07.018)
Supplement: Supplementary file 1 — Supplementary Material [file mmc1.pdf]

A Neuro-Computational Account of How Inflammation Enhances Sensitivity to Punishments Versus Rewards

Supplemental Information

SUPPLEMENTAL METHODS & MATERIALS

Bayesian Model Selection

Participants' mean EPI images were first segmented then normalized to a 3 mm MNI template. Normalized (unsmoothed) EPI volumes were then used in two alternate first-level Bayesian models in SPM12 and model evidence maps computed (1). The first model (Model 1) was that reported in the original paper, the second model (Model 2) included an outcome value parametric regressor ([0 1] in the gain condition, [0 -1] in the loss condition) instead of the PE parametric regressor. Both models included subject-specific realignment parameters that were modeled as covariates of no interest to correct for motion artifacts. To ensure computation within a reasonable timeframe, model estimation was restricted to the bilateral ventral striatum and right anterior insula regions of interest reported in the main paper. Activation maps for reward (rPE) and punishment prediction error (pPE) in ventral striatum and anterior insula reported in Pessiglione's original paper (2) using this task were used to produce this region of interest mask.

A random effects second level Bayesian model selection was then performed using the SPM12 function BMS-maps (inference), with the log evidence maps for each of the two separate models entered for each participant (3). Results for exceedance probabilities were then obtained using the SPM12 BMS-maps (results) function.

SUPPLEMENTAL RESULTS

Bayesian Model Selection (BMS)

BMS confirmed that Model 1 (the original model using PE regressor at the time of outcome) showed higher exceedance probability for the right insula (0.881 versus 0.119 for Model 2)

(Figure S1A). This result held for both the whole right insula (using the mask for pPE obtained from the original Pessiglione paper), as well as specifically for the right insula region (Figure S1C) in which we demonstrate a significant increase in pPE following inflammation in the main paper (exceedance probability 0.881 versus 0.119).

The findings for the ventral striatum (VS) were more complex. Taking the VS region as a whole (using a mask for rPE obtained from the original Pessiglione paper), Model 2 (outcome value) had a higher exceedance probability than Model 1 (0.119 versus 0.881 for Model 2) (Figure S1B). However, when we looked specifically at the right VS sub-region in which we report a significant decrease in rPE following inflammation (Figure S1C) our original model (Model 1) showed the greater exceedance probability (0.731 versus 0.269 for Model 2) (Figure S1D).

SUPPLEMENTAL DISCUSSION

These Bayesian model selection analyses support an effect of inflammation that is mediated via actions on neural representations of reward and punishment prediction error. This is shown most clearly for pPE in which Model 1 has higher exceedance probabilities for both the whole right insula (using the mask for pPE obtained from the original Pessiglione paper), as well as specifically for the right insula region (Figure S1C) in which we demonstrate a significant increase in pPE following inflammation in the main paper. However, the additional new finding that much of the ventral striatum activity (but interestingly not that region showing a significant effect of inflammation) can be better explained by modeling outcome value (rather than PE) will need to be explored in future studies.

Table S1. Significant clusters observed for processing cues

Side	Region	Coordinates	*Z* Score	k	*p*	FWE (ROI)
			**Gain minus Neutral Cues**			
L	Ventral Striatum[a]	[-8 10 0]	4.79	90	<0.001	0.256 (0.001)
R	Ventral Striatum[a]					n/a
			**Loss minus Neutral Cues**			
R	Anterior Insula[a]	[36 22 -8]	4.29	193	<0.001	0.023 (0.001)
L	Anterior Insula[a]	[-30 22 -8]	3.92	49	<0.001	0.657 (0.001)
L	Precuneus	[-8 -70 46]	5.56	715	<0.001	0.001
L	Dorsal Striatum	[-12 10 8]	5.21	254	<0.001	0.006
R	Dorsal Striatum	[12 8 10]	4.67	241	<0.001	0.008
R	DLPFC	[-42 6 46]	5.12	364	<0.001	0.001
L	DLPFC	[44 12 42]	4.30	369	<0.001	0.001
L	Parietal	[-40 -52 54]	4.35	269	<0.001	0.005
R	Parietal	[36 -60 54]	4.19	331	<0.001	0.001

Only clusters surviving whole brain or region of interest (reported in brackets) family wise error (FWE) correction are reported. K denotes cluster extent, [x y z] are MNI coordinates.

[a] denotes a priori regions of interest.

Table S2. Effects of inflammation on reward & punishment prediction error

Side	Region	Coordinates	*Z* Score	k	*p*	FWE (ROI)
			**Reward: Vaccine < Placebo**			
L	Ventral Striatum[a]	n/a	n/a	0	<0.05	n/a
R	Ventral Striatum[a]	[8 2 2]	3.55	88	<0.05	(0.021)
			**Punishment: Placebo > Vaccine**			
L	Anterior Insula[a]	[28 28 -8]	3.50	412	<0.05	(0.026)

Only clusters surviving whole brain or region of interest (reported in brackets) family wise error (FWE) correction are reported. k denotes cluster extent, [x y z] are MNI coordinates.

[a] denotes a priori regions of interest.

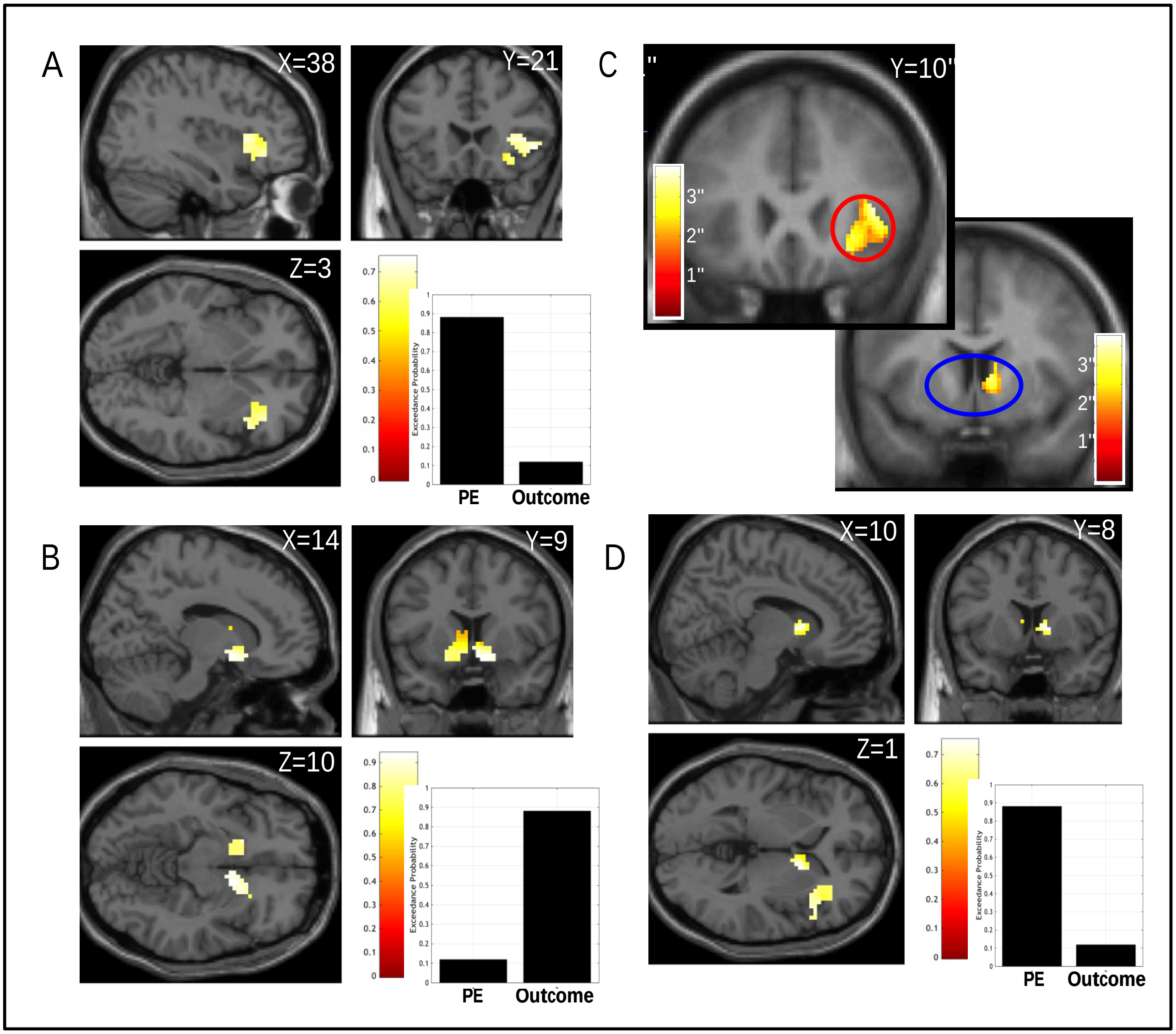

Figure S1. Bayesian Model Selection. (**A**) Exceedance probabilities for the whole right insula region of interest for Model 1 (PE model) and Model 2 (Outcome value model). (**B**) Exceedance probabilities for the whole ventral striatal region of interest for Model 1 (PE model) and Model 2 (Outcome value model). (**C**). Right anterior insula (red circle) and ventral striatal (blue circle) regions showing significant effect of inflammation on punishment and reward prediction error respectively. (**D**) Exceedance probabilities for the right ventral striatal region showing an effect of inflammation on reward prediction error encoding for Model 1 (PE model) and Model 2 (Outcome value model).

Supplemental References

1. Rosa MJ, Bestmann S, Harrison L, Penny W (2010): Bayesian model selection maps for group studies. *NeuroImage* 49: 217-224.
2. Pessiglione M, Seymour B, Flandin G, Dolan RJ, Frith CD (2006): Dopamine-dependent prediction errors underpin reward-seeking behaviour in humans. *Nature* 442: 1042-1045.
3. Stephan KE, Penny WD, Daunizeau J, Moran RJ, Friston KJ (2009): Bayesian model selection for group studies. *Neuroimage* 46: 1004-1017.
